# Comparison of the Influence of Dual-Task Activities on Prefrontal Activation and Gait Variables in Older Adults with Mild Cognitive Impairment during Straight and Curved Walking

**DOI:** 10.3390/medicina60020235

**Published:** 2024-01-29

**Authors:** Yumin Lee, Jihye Jung, Hyunjoong Kim, Seungwon Lee

**Affiliations:** 1 Department of Physical Therapy, Graduate School, Sahmyook University, 815 Hwarang-ro, Seoul 01795, Republic of Korea; ljm200660@naver.com; 2 Institute of SMART Rehabilitation, Sahmyook University, 815 Hwarang-ro, Nowon-gu, Seoul 01795, Republic of Korea; jihye3752@gmail.com; 3 Neuromusculoskeletal Science Laboratory, 15 Gangnam-daero 84-gil, Seoul 06232, Republic of Korea; hyun-joongkim@nmslab.org; 4 Department of Physical Therapy, Sahmyook University, 815 Hwarang-ro, Seoul 01795, Republic of Korea

**Keywords:** mild cognitive impairment, dual-task training, functional near-infrared spectroscopy, walking speed, step count

## Abstract

*Background and Objectives*: Mild cognitive impairment (MCI) is an early stage of dementia in which everyday tasks can be maintained; however, notable challenges may occur in memory, focus, and problem-solving skills. Therefore, motor-cognitive dual-task training is warranted to prevent cognitive decline and improve cognition in aging populations. This study aimed to determine the influence of such dual-task activities during straight and curved walking on the activities of the prefrontal cortex and associated gait variables in older adults with MCI. *Materials and Methods*: Twenty-seven older adults aged ≥65 years and identified as having MCI based on their scores (18–23) on the Korean Mini-Mental State Examination were enrolled. The participants performed four task scenarios in random order: walking straight, walking straight with a cognitive task, walking curved, and walking curved with a cognitive task. The activation of the prefrontal cortex, which is manifested by a change in the level of oxyhemoglobin, was measured using functional near-infrared spectroscopy. The gait speed and step count were recorded during the task performance. *Results*: Significant differences were observed in prefrontal cortex activation and gait variables (*p* < 0.05). Specifically, a substantial increase was observed in prefrontal cortex activation during a dual task compared with that during a resting-state (*p* < 0.013). Additionally, significant variations were noted in the gait speed and step count (*p* < 0.05). *Conclusions*: This study directly demonstrates the impact of motor-cognitive dual-task training on prefrontal cortex activation in older adults with MCI, suggesting the importance of including such interventions in enhancing cognitive function.

## 1. Introduction

Mild cognitive impairment (MCI), a precursor to dementia, is characterized by a slight decline in cognitive functions, such as thinking, judgment, language, and memory. Although individuals with MCI can typically perform daily activities independently, they may encounter difficulties in performing more complex tasks such as handling banking matters [1,2,3]. MCI frequently results in gait disorders, given that physical functionality, motor accuracy, balance, and spatial cognition are generally lower than those in healthy older adults [4]. This decline can pose challenges for individuals with MCI in maintaining stability or responding to questions while walking, thereby increasing the risk of falls and unstable posture [5,6]. Gait speed is also associated with executive function in older adults with MCI, and a decreased gait speed is associated with a reduced cognitive processing speed [7,8].

Recent studies have actively investigated the cognitive health and brain activity in older adults, with a particular focus on the relationship between the cerebral blood flow and cognitive function [9]. The high energy consumption in specific brain regions owing to neuronal activity leads to an increase in cerebral blood flow. This flow is crucial for maintaining normal brain function, as it supplies oxygen and nutrients to the brain cells and aids in waste removal [10,11]. The link between cerebral blood flow and cognitive function is demonstrated by the concentration of oxyhemoglobin (HbO_2_) in the prefrontal cortex (PFC), as observed through functional magnetic resonance imaging (fMRI) [9,12,13]. Notably, the cerebral blood flow in individuals with dementia and MCI is reduced during rest compared with that in individuals with normal cognitive function [14,15]. Functional near-infrared spectroscopy (fNIRS) examination has shown that during the performance of cognitive tasks [16], the HbO_2_ levels in the PFC of individuals with MCI are lower than those of healthy and older adults [17]. This reduction in the prefrontal HbO_2_ level, which is involved in various cognitive functions such as attention, working memory, and problem solving, indicates a decrease in brain activity, a phenomenon also observed in the pre-dementia stage of MCI [17,18,19].

To delay the progression of MCI to dementia, cognitive training, including single or dual tasks (e.g., cognitive motor training), can be applied [20]. These include cognitive cycling training [21], virtual reality-based cognitive motor training [3,22], and sensory stimulation training using visual or auditory cues [23]. Although most dual-task interventions are effective, their implementation can be costly or depend on the location, thus limiting accessibility. Cognitive walking training overcomes these spatial and economic limitations, and walking-cognitive training has been recommended [5]. Although most studies on dual-task walking have focused on straight walking, walking in straight lines or in simple environments may not fully replicate the demands of walking in complex settings [24]. In real-life situations, navigating curved paths or maneuvering around obstacles requires adjusting one’s path, changing direction, and maintaining a balance [25,26]. Therefore, curved walking, which demands greater attention and working memory compared with straight walking, could play a significant role in training for stable and flexible gait, particularly in high-risk fall situations [26,27].

Accordingly, cognitive walking training is anticipated to provide various benefits by simultaneously improving the cognitive and walking abilities of individuals with MCI. This study aimed to explore the effects of dual-task performance during straight and curved walking on prefrontal cortex activation and gait variables in older adults with MCI.

## 2. Materials and Methods

### 2.1. Study Design

This observational cross-sectional study was carried out in accordance with the STrengthening the Reporting of OBservational studies in Epidemiology (STROBE) guidelines [28]. This study was approved by the Ethics Committee of Sahmyook University prior to the commencement of the experiment (date: 13 February 2023 and no. SYU 2023-02-013-002). Additionally, the study was pre-registered and approved by ClinicalTrials.gov (date: 2 June 2023 and no. NCT05885867).

### 2.2. Participants

Community-dwelling older adults aged ≥65 years who were capable of performing dual tasks were considered eligible for the study. Participants who scored 18–23 points on the Korean version of the Mini-Mental State Examination (K-MMSE) ver. 2, were considered MCI [2], and were able to walk using assistive devices were included [29]. The individuals were unable to walk 3 m forward and backward or get up from a seated position, showed sensitive reactions to near-infrared lasers [30], had visual and hearing impairments [9,31,32], and had a history of severe neurological diagnoses or depression [33].

### 2.3. Sample Size

The required sample size was calculated using G*power software (version 3.1, University of Kiel, Kiel, Germany). To evaluate the effectiveness of the cognitive-motor dual tasks, parameters were set based on the findings of a previous study [31]: repeated-measures analysis of variance, a significance level of 0.05, a power of 0.95, and an effect size of 0.3. Based on these calculations, a minimum of 26 participants were required. Considering an anticipated dropout rate of approximately 15%, 30 participants were recruited for this study.

### 2.4. Procedures

The participants were recruited through verbal promotions. All potential participants who met the selection criteria were thoroughly informed of the purpose and methods of the study, and only those who voluntarily signed a consent form participated in the experiment.

Ultimately, 28 older adults with MCI who wish to participate and perform four different tasks were included. Initially, the participants’ general characteristics were collected. Before performing the tasks, a device capable of measuring PFC activation was placed on each participant’s forehead. The participants were seated in a comfortable position. After wearing the fNIRS and zero calibration device, the resting-state activation of the PFC was evaluated. Following a 30 s rest, the participants performed single (motor) and dual (motor-cognitive) tasks in straight and curved paths while wearing the fNIRS device. Various tasks were randomly assigned, and each task was measured three times to calculate the average values. PFC activation was measured during task performance, along with the walking speed and step count. All variables were measured three times and averaged for use.

#### Single Task and Dual Task

**Figure 1:**
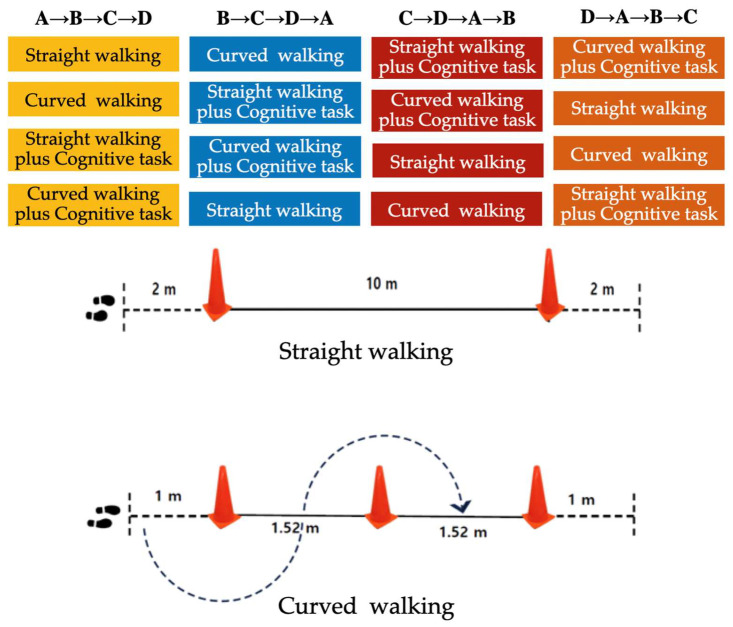
Single-task and dual-task procedures.

The tasks in this study comprised four variations: straight walking (A), curved walking (B), straight walking with a cognitive task (C), and curved walking with a cognitive task (D). The sequence of tasks was arranged in the following order: A → B → C → D, B → C → D → A, C → D → A → B, and D → A → B → C (Figure 1). The cognitive task of counting backwards from 100 was applied equally to straight and curved walking. The participants were instructed to walk at their normal pace; if incorrect answers were provided during the performance of cognitive tasks, correct numbers were provided, and errors were marked for further review. To ensure accurate measurements, a minimum rest period of 30 s was allowed between tasks [34]. Additionally, considering the age of the participants, sufficient rest was provided upon request, and a physical therapist was assigned to assist with falls and safety.

Straight walking was measured using the 10 m walk test (10 MWT). The total distance was set at 14 m, with 2 m set at the start and end to allow for acceleration and deceleration (Figure 1). The participants were asked to walk at a normal pace [35]. Curved walking was measured using a modified figure-of-eight walk test (mF8WT), adapted specifically for this study. The distance between the three cones was set to 1.52 m each, and the participants were instructed to avoid deviating within >1 m from the cones when turning [6] (Figure 1). The participants were requested to discontinue the performance of the task if dizziness or any changes in their physical condition occurred. In such cases, the participants were allowed to sit on a chair placed nearby. The participants were also instructed to walk under normal conditions [35].

### 2.5. Outcomes

#### 2.5.1. Prefrontal Cortex Activation

**Figure 2:**
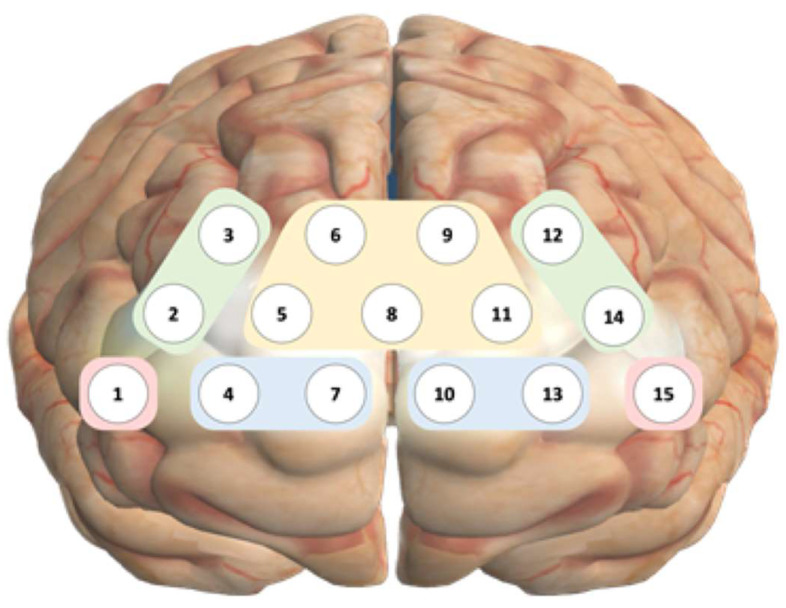
Location of cortical regions by channel. 1, Right lateral orbitofrontal cortex; 2 and 3, right dorsolateral prefrontal cortex; 4 and 7, right medial orbitofrontal cortex; 10 and 13, left medial orbitofrontal cortex; 12 and 14, left dorsolateral prefrontal cortex; 15, left lateral orbitofrontal cortex; 5, 6, 8, 9, and 11, frontopolar prefrontal cortex.

In this study, NIRSIT Lite (OBELAB Inc., Seoul, Republic of Korea), an fNIRS device, was used to measure PFC activation. This device consists of 15 channels, each separated by a distance of 30 mm between pairs. Among these, channels 1–7 were located on the right PFC, channel 8 was positioned at the center, and channels 9–15 were arranged symmetrically on the left PFC. The channels were further divided into four regions: the lateral, medial, posterior, and frontopolar PFC (Figure 2). The device, equipped with five dual-wavelength (780/850 nm) laser sources and seven detectors, measures the light diffused within a tissue volume according to the light propagation model. Light can reach approximately 8 mm into the cortical surface with a source-detector separation of 3 cm [36].

Before performing the tasks, the changes in HbO_2_ levels were measured during a 30 s rest period while the respective task was carried out.

#### 2.5.2. Gait Speed

The walking speed of the participants was measured using a stopwatch, taking into account the acceleration and deceleration over a straight 14 m path and a curved path. The measurements obtained at the initial and final 2 m were excluded to account for the changes in speed. The walking speed was calculated by dividing the walking distance by the time [37]. The test–retest reliability of walking speed measurements in older adults has an intraclass correlation coefficient of 0.96–0.99 [38].

#### 2.5.3. Steps

The number of steps taken was measured using a handheld manual counter during the 10 MWT and mF8WT [39]. The start and end points of the lines were marked, and only the steps taken within these lines were counted; steps outside the lines were not included.

### 2.6. Data Analysis

Statistical analysis of the data was conducted using SPSS ver. 25.0 software for Windows (SPSS Inc., Chicago, IL, USA), from which the means and standard deviations were derived. A normality test was conducted for all participants, and descriptive statistics were used to characterize their general characteristics. A repeated-measures analysis of variance was conducted to determine the differences in PFC activation, walking speed, and step count among the participants. To compare the differences between straight and curved walking under various conditions, a paired *t*-test was performed; meanwhile, an independent *t*-test was used to compare the differences in PFC activation between the cerebral hemispheres. The significance level was set at a *p* value of 0.05. Furthermore, to examine the differences between each task condition, a Bonferroni correction was applied, setting the significance level at α = 0.013.

## 3. Results

**Figure 3:**
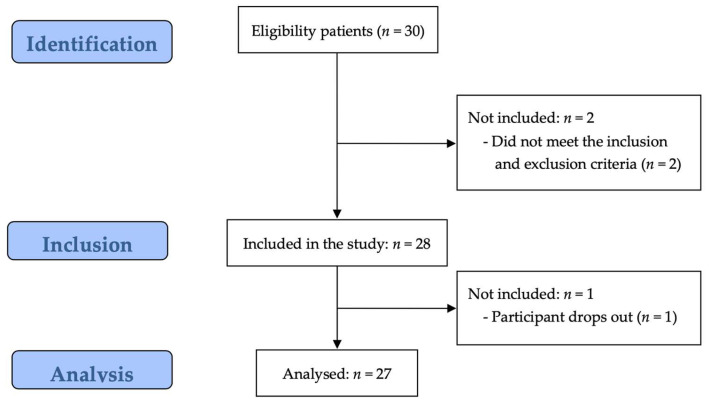
Flow chart showing the study process following the strengthening of the reporting of observational studies’ guidelines.

Figure 3 presents a flow diagram of this study based on the STROBE guidelines. Thirty potential participants underwent eligibility screening, and two were excluded. Finally, 28 enrolled participants were assessed. However, 1 dropped out of the study, leaving 27 participants for the analysis.

### 3.1. Characteristics of the Enrolled Participants

The study included 27 participants (9 men and 18 women) with an average age of 80.15 years. The average height was 157.19 cm, while the average weight was 58.15 kg. The mean K-MMSE score was 22.26 points (Table 1).

### 3.2. Comparison of the Activity of the Prefrontal Cortex According to the Cerebral Hemisphere

When the activation of the PFC in the cerebral hemispheres was compared, significant differences were found between the right and left hemispheres in the posterior PFC area during straight walking (*p* < 0.05); however, no significant differences were observed during straight walking with a cognitive task, curved walking, or curved walking with a cognitive task (*p* > 0.05). No significant differences were found between the right and left hemispheres in the frontopolar PFC during straight walking, straight walking with a cognitive task, curved walking, or curved walking with a cognitive task (*p* > 0.05).

### 3.3. Comparison with the Resting State

When the activation of the PFC was compared during rest and single tasks, significant differences were found in channels 4, 9, and 11 during straight walking (*p* < 0.013) and in channel 6 during curved walking (*p* < 0.013).

**Figure 4:**
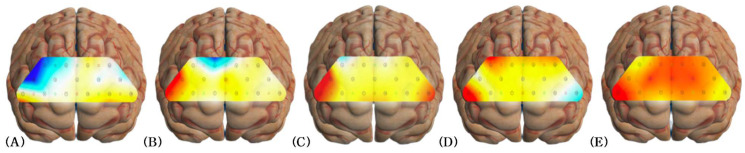
Changes in prefrontal cortex activation. (**A**) At rest; (**B**) straight walking; (**C**) straight walking + cognitive task; (**D**) curved walking; (**E**) curved walking + cognitive task. Red indicates high levels of activation; blue indicates low levels of activation.

When the activation of the PFC was compared during rest and motor-cognitive dual tasks, significant differences were observed in channels 5, 7, 9, and 10 during straight walking with a cognitive task (*p* < 0.013) and in channels 1, 4, 5, 7, 8, 10, 13, and 14 during curved walking with a cognitive task (*p* < 0.013) (Table 2) (Figure 4).

### 3.4. Comparison of Straight and Curved Walking

When the activation of the PFC was compared during straight walking and curved walking, significant differences were found in channels 5, 9, and 11 (*p* < 0.05). When straight walking with a cognitive task was compared to curved walking with a cognitive task, a significant difference was found in channel 10 (*p* < 0.05) (Table 2).

### 3.5. Changes in Gait Speed Depending on Conditions

The changes in gait speed under various conditions were as follows: straight walking at 0.88 ± 0.36 m/s, straight walking with a cognitive task at 0.70 ± 0.31 m/s, curved walking at 0.68 ± 0.28 m/s, and curved walking with a cognitive task at 0.50 ± 0.24 m/s, with significant differences observed between each condition (*p* < 0.05) (Table 3). The step count changes under various conditions were as follows: straight walking at 19.00 ± 3.11 steps, straight walking with a cognitive task at 19.63 ± 4.12 steps, curved walking at 21.48 ± 4.64 steps, and curved walking with a cognitive task at 22.52 ± 5.42 steps, with significant differences observed between conditions (*p* < 0.05) (Table 3).

## 4. Discussion

This cross-sectional study investigated the effects of dual-task performance during straight and curved walking on PFC activation and gait variables in older adults with MCI. The decreased activation of the PFC, which is associated with various cognitive functions, may increase the incidence of MCI in older adults [17]. The results of this study revealed significant differences in PFC activation during dual-task performance, walking speed, and step count in older adults with MCI.

In this study, significant differences were found in PFC activation in 8 of the 15 channels in various conditions (*p* < 0.05). The activated channels included the right lateral PFC (channel 1), right posterior PFC (channel 2), right medial PFC (channel 4), right medial PFC (channels 5 and 6), central PFC (channel 8), and left PFC (channels 9 and 11). The right lateral PFC is associated with cognitive and individual decision-making, emotions, and the reward system and plays an important role in social interaction and adaptation [40]. The right posterior PFC is involved in cognitive control processes and regulates working memory, reasoning, and planning. Its involvement enhances cognitive flexibility and the accuracy and efficiency of problem solving [41]. The right medial PFC plays a critical role in regulating emotions, facilitating social interaction, motivating behavior, and reward processing. As a result, it significantly influences human social behavior and emotional experiences [42]. The frontopolar PFC is associated with higher-order cognitive processes and regulates interactions with other brain regions. It also integrates and regulates functions related to information processing, working memory, cognitive flexibility, and attention, thereby supporting complex cognitive processes [43].

This study analyzed the differences in activation between the cerebral hemispheres. A significant difference was observed in the right hemisphere of the posterior PFC during straight walking compared with the left hemisphere (*p* < 0.05). Although no significant differences were found during straight walking with a cognitive task, curved walking, or curved walking with a cognitive task (*p* > 0.05), differences in PFC activation were noted in the right hemisphere compared with the left hemisphere. The right posterior PFC, which is primarily involved in spatiotemporal tasks and in regulating working memory and cognitive flexibility [44], is believed to be activated. No significant differences were observed between the right and left hemispheres in the frontopolar PFC during straight walking (*p* > 0.05). Although no significant differences were observed during straight walking with a cognitive task, curved walking, or curved walking with a cognitive task (*p* > 0.05), differences in PFC activation were noted in the right hemisphere compared with the left hemisphere. This finding is similar to those of previous studies, which indicated that the right PFC, responsible for visual information processing and spatial cognition regulation, becomes hyperactivated to compensate for language processing and cognitive tasks in older adults with MCI [45]. No significant differences were found between the left and right hemispheres, possibly because the frontopolar PFC channels are centrally located and are included in the overall activated channels. Future studies should analyze the cerebral hemispheres separately to verify the changes in activation with greater accuracy.

In this study, the differences in activation according to the condition were analyzed using the measurements obtained at rest as the baseline. Significant differences were observed in 11 of the 15 channels during the performance of the four tasks compared with that at rest (*p* < 0.013). The activated channels included the right lateral PFC (channel 1), medial PFC (channels 4, 7, 10, and 13), frontopolar PFC (channels 5, 6, 8, 9, and 11), and left posterior PFC (channel 14). This observation is consistent with that of a previous study, which suggested that resting activation of the PFC in older adults with MCI decreases and activation increases during dual tasks, such as walking while reading or speaking [9,46]. This study suggests that dual-task performance impacts behavior and thought as it improves cognitive and social emotions, problem-solving abilities, and interactions with other brain areas [43,47]. These findings predict that cognitive abilities are maintained as HbO_2_ levels decrease during rest and increase when attention, working memory, and problem-solving abilities are required during dual-task performance. These findings could be utilized in future studies on cognitive enhancement [33].

This study also analyzed the differences in activation according to the walking method. More significant differences were observed in the three channels during curved walking and in one channel during curved walking with a cognitive task compared with straight walking (*p* < 0.05). Activated channels were located in the frontopolar PFC (channels 5, 6, 9, and 11). Curved walking requires more cognitive processing and attention than straight walking because of the need to fine-tune the asymmetric foot placement and direction changes [27]. Performing new motor patterns and movements can help form and strengthen neural connections in the brain, which are linked to improved cognitive function and increased brain activity in older adults [48]. Thus, this study suggests that dual-task performance during curved walking showed enhanced activation in the frontopolar PFC compared with straight walking. However, as a prior assessment of the participants’ walking abilities was not conducted, individual differences in walking ability may have influenced the results. Considering these limitations, future research should assess the participants’ walking abilities to increase the accuracy of the findings.

The study found that channels located in the frontopolar PFC area were activated the most (approximately 14.29% more than the other channels). The frontopolar PFC, which regulates cognitive function and processes complex information, exhibits increased activation as it receives more information during dual tasks than during rest [49]. Therefore, performing dual tasks in daily life requires interaction with various cognitive functions that can help maintain functionality and focus on cognitive tasks.

Additionally, the study analyzed the differences in gait variables employed in previous studies that used dual tasks to evaluate the interactions between cognition, gait, and fall risk [5]. The cognitive task in this study, counting backwards, will adapt the easy level of Pardhan and Zuidhoek [50]. Counting backwards is a cognitive function that requires working memory and attention. The study showed that walking speed significantly decreased during straight and curved walking while counting numbers backward (*p* < 0.05). This finding is consistent with those of previous studies showing a progressive decrease in walking speed during the performance of complex cognitive tasks, such as subtracting 7 from 100 [5]. Older adults may experience walking disorders when performing additional complex tasks related to attention or memory due to cognitive decline [51]. Therefore, the walking speed decreases and the step count increases owing to the inability to perform natural walking motions [52]. Hence, intervention studies should be conducted to assess the changes in walking over time, taking into account the correlation between walking variables and cognitive decline [53].

Previous studies on older adults have shown that cognitive flexibility and posture transition processes contribute to curved walking, providing different information compared with straight walking [24]. In addition, studies conducted among older adults with MCI who required the performance of dual tasks reported an increased activation in the PFC; however, these findings were observed during straight walking [33]. However, this study simultaneously evaluated the increase in PFC activation during dual-task performance and curved walking to examine how these two factors interact to affect walking performance. Based on these results, this study emphasizes the benefits of curved walking, showing that dual-task performance during curved walking may provide superior outcomes.

This study had several limitations. First, brain activation during task performance could have been influenced by the task difficulty and cognitive function levels of the participants [33]. Therefore, future studies should perform individualized cognitive tasks tailored to the participants’ cognitive functions. Second, the study was conducted only in older adults with MCI; therefore, the differences in brain activation during the task performance between older adults with MCI and healthy older adults were not assessed. Hence, studies comparing the PFC activation between healthy older adults and those with MCI should be conducted. Finally, this was a cross-sectional study conducted over a short period, making it difficult to observe any improvements in cognitive function. Therefore, future intervention studies should be conducted to determine whether the activation of the PFC during dual tasks can improve cognitive function in patients with MCI.

## 5. Conclusions

Through this study, we can understand the relationship between cognitive tasks and PFC activation and propose methods for improving cognitive abilities in older adults with MCI through simple training. Such methods could aid in the prevention and management of cognitive decline in older adults, allowing for functional improvements in daily life. Furthermore, with the aging of the population, these approaches could contribute to enhancing the quality of life and health of older adults and play an important role in the prevention and management of dementia.

## Figures and Tables

**Table 1:** Characteristics of the enrolled participants.

	Mean ± SD
Sex (male/female)	9/18
Age (years)	80.15 ± 6.27
Height (cm)	157.19 ± 7.62
Weight (kg)	58.15 ± 9.16
K-MMSE (point)	22.26 ± 1.56

Note. K-MMSE, Korean-mini mental state examination.

**Table 2:** Prefrontal cortex activation.

Channel (μM/mm)	Resting	Straight Walking	Straight Walking Plus Cognitive Task	Curved Walking	Curved Walking Plus Cognitive Task	F (*p*)
1	−0.43 ± 1.42	0.19 ± 1.51	0.15 ± 1.23	0.49 ± 1.54	0.88 ± 1.69 ^†^	2.875 (0.026) *
2	0.05 ± 0.87	0.52 ± 0.79	0.73 ± 1.29	−0.09 ± 1.39	0.66 ± 1.34	2.559 (0.043) *
3	−0.09 ± 0.60	0.47 ± 1.06	0.12 ± 1.26	0.29 ± 0.81	0.55 ± 1.43	1.770 (0.141)
4	−0.20 ± 0.77	0.41 ± 0.50 ^†^	0.55 ± 1.06	0.22 ± 0.88	0.79 ± 1.29 ^†^	4.137 (0.004) *
5	−0.23 ± 0.78	0.42 ± 0.63	0.65 ± 1.23 ^†^	−0.06 ± 0.89 ^‡^	0.65 ± 1.45 ^†^	3.079 (0.038) *
6	−0.12 ± 0.57	0.50 ± 1.08	0.35 ± 0.61	0.28 ± 0.75 ^†^	0.48 ± 1.06	2.702 (0.035) *
7	−0.19 ± 0.58	0.43 ± 1.08	0.37 ± 0.77 ^†^	0.19 ± 0.62	0.66 ± 1.20 ^†^	2.125 (0.113)
8	−0.56 ± 1.71	0.47 ± 1.61	0.58 ± 1.20	−0.03 ± 1.27	0.68 ± 1.84 ^†^	3.091 (0.020) *
9	−0.11 ± 0.50	0.65 ± 0.73 ^†^	0.56 ± 0.97 ^†^	0.03 ± 0.93 ^‡^	0.66 ± 1.20	4.133 (0.004) *
10	−0.34 ± 1.37	0.53 ± 1.07	0.28 ± 0.79	0.12 ± 0.7	0.68 ± 0.96 ^†,‡^	2.131 (0.117)
11	−0.15 ± 0.80	0.65 ± 0.86	0.89 ± 0.87 ^†^	0.04 ± 0.65 ^‡^	0.58 ± 1.89	3.100 (0.039) *
12	−0.11 ± 0.79	0.72 ± 1.02 ^†^	0.23 ± 0.69	0.25 ± 0.95	0.02 ± 2.10	2.089 (0.120)
13	−0.04 ± 0.47	0.48 ± 0.75	0.35 ± 0.67	0.34 ± 0.78	0.69 ± 1.25 ^†^	2.446 (0.084)
14	−0.15 ± 0.87	0.52 ± 0.87	0.54 ± 0.83	0.21 ± 1.13	0.57 ± 1.30 ^†^	2.692 (0.062)
15	−0.30 ± 0.97	0.15 ± 0.86	0.18 ± 0.66	0.09 ± 1.31	0.55 ± 0.99	2.339 (0.092)

Note. 1, right lateral orbitofrontal cortex; 2 and 3, right dorsolateral prefrontal cortex; 4 and 7, right medial orbitofrontal cortex; 10 and 13, left medial orbitofrontal cortex; 12 and 14, left dorsolateral prefrontal cortex; 15, left lateral orbitofrontal cortex; 5, 6, 8, 9, and 11, frontopolar prefrontal cortex. The values are presented as the mean ± SD. * Significant difference in prefrontal cortex activation by region (*p* < 0.05). ^†^ Significant difference between the resting state and task condition (*p* < 0.013 ^‡^). Significant difference between straight walking and curved walking (*p* < 0.05).

**Table 3:** Changes in gait speed depending on the condition.

	Straight Walking	Straight Walking Plus Cognitive Task	Curved Walking	Curved Walking Plus Cognitive Task	F (*p*)
Gait speed (m/s)	0.88 ± 0.36	0.70 ± 0.31	0.68 ± 0.28	0.50 ± 0.24	34.735 (0.000) *
Steps	19.00 ± 3.11	19.63 ± 4.12	21.48 ± 4.64	22.52 ± 5.42	4.4375 (0.013) *

Note. The values are presented as the mean ± SD. * *p* < 0.05.

## Data Availability

Data are contained within the article.
